# Magnetic compression anastomosis for recanalization of anastomotic stenosis after radical surgery for esophageal cancer

**DOI:** 10.1055/a-2512-7680

**Published:** 2025-01-28

**Authors:** Miaomiao Zhang, Huanchen Sha, Guifang Lu, Mudan Ren, Shuixiang He, Yi Lv, Xiaopeng Yan

**Affiliations:** 1 162798 Department of Hepatobiliary Surgery, The First Affiliated Hospital of Xiʼan Jiaotong University, Xiʼan, China; 2 162798 Shaanxi Provincial Key Laboratory of Magnetic Medicine, The First Affiliated Hospital of Xiʼan Jiaotong University, Xiʼan, China; 3 162798 Department of Gastroenterology, The First Affiliated Hospital of Xiʼan Jiaotong University, Xiʼan, China

A 67-year-old man who had previously undergone radical surgery for esophageal cancer had experienced dysphagia over the past year. Gastroscopy revealed esophageal anastomotic stenosis. Multiple endoscopic balloon dilation and esophageal stent placement procedures were carried out, with limited success. Following reconfirmation of the stenosis through gastroscopy and esophagography ( Fig. 1 ), a treatment plan utilizing magnetic compression anastomosis (MCA) was devised ( Fig. 2 ).

**Fig. 1:**
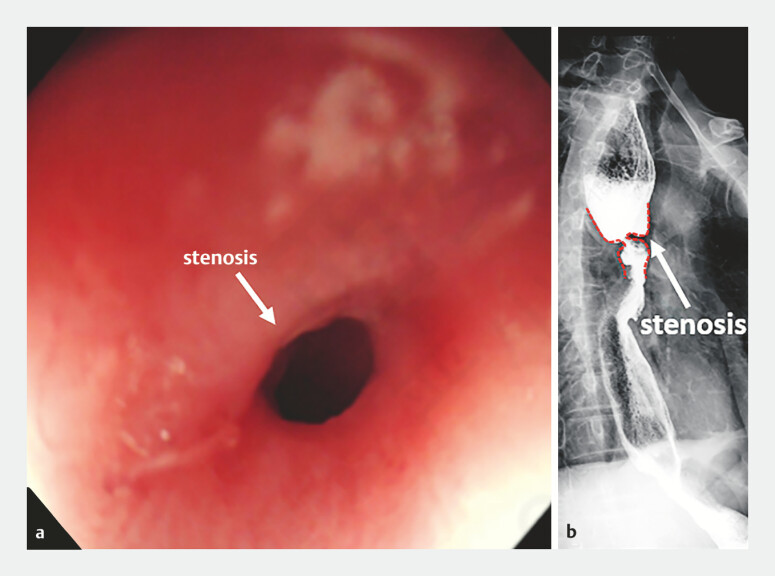
Esophageal stenosis confirmed: **a** gastroscopy; **b** esophagography.

**Fig. 2:**
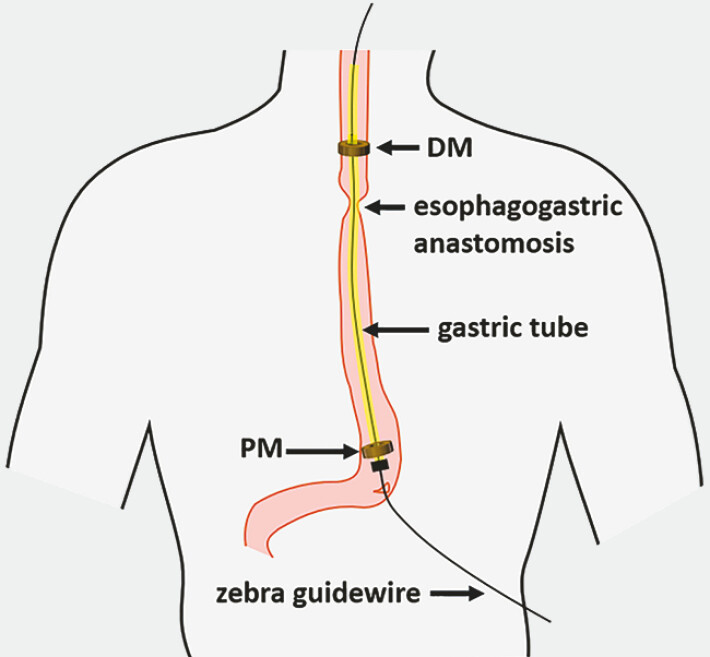
Schematic diagram of the surgical procedure.

Following induction of anesthesia, an incision was made in the anterior wall of the stomach under laparoscopy (Olympus Corporation, Tokyo, Japan). A gastroscope (Olympus Corporation, Tokyo, Japan) was introduced through the mouth and advanced to the stenosis, where a zebra guidewire (Jiangsu Vedkang Medical Science and Technology Co., Ltd., Changzhou, China) was inserted via the biopsy channel to traverse the stenosis and enter the stomach. The zebra guidewire was subsequently pulled out through the incision on the stomach and exited via the trocar. The parent magnets (PM) (Shaanxi Fengyuan Zhongjia Electronic Technology Co., Ltd., Xianyang, China) were inserted through the tail of the gastric tube, after which a zebra guidewire was inserted into the gastric tube ( Fig. 3 **a** ). The gastric tube and PM were pushed into the abdominal cavity and continued to be pushed after passing through the gastric incision. The PM were pulled until they reached the distal end of the stenosis ( Fig. 3 **b** ). The daughter magnets (DM) (Shaanxi Fengyuan Zhongjia Electronic Technology Co., Ltd., Xianyang, China) were then inserted into the head of the gastric tube, and a tube was used to push them into the proximal end of the stenosis ( Fig. 3 **c** ). X-Radiography confirmed adherence of the magnets through attraction ( Fig. 3 **d** ).

**Fig. 3:**
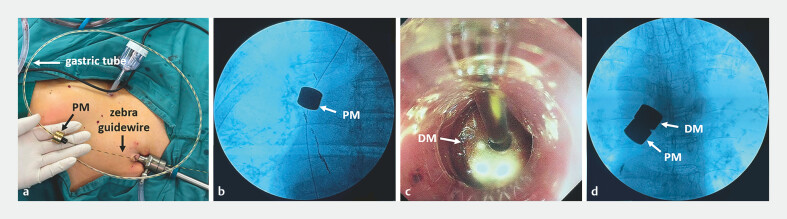
The surgical procedure: **a** the parent magnets (PM) were inserted into the gastric tube; **b** the PM were pulled to the distal end of the stenosis; **c** the daughter magnets (DM) were inserted along the gastric tube into the proximal end of the stenosis; **d** the PM and DM adhering together through attraction.

Esophagography was conducted regularly after surgery to assess the establishing of the anastomosis ( Fig. 4 ). After 19 days, the magnets were removed and an esophageal stent (Micro-Tech [Nanjing] Co., Ltd., Nanjing, China) was inserted ( Fig. 5 , Video 1 ). To date, 7 months after the magnets were removed, the patient has been able to eat by mouth without difficulty.

**Fig. 4:**
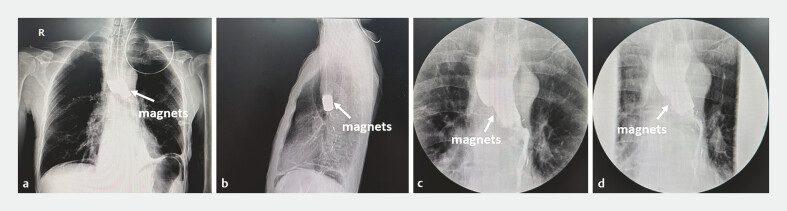
Postoperative X-ray monitoring by esophagography: **a, b** postoperative day 2; **c** postoperative day 12; **d** postoperative day 16.

**Fig. 5:**
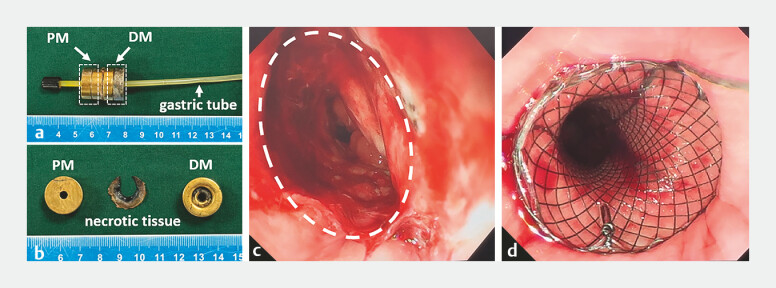
Establishing of magnetic compression anastomosis: **a, b** the magnets removed 19 days after surgery; **c** the anastomosis as seen on endoscopy; **d** the esophageal stent in place.

Magnetic compression anastomosis for recanalization of anastomotic stenosis after radical surgery for esophageal cancer.Video 1

The combination of MCA and endoscopy presents a viable treatment option for various types of gastrointestinal obstruction 1 2 3 4 5 . This report introduces a novel treatment approach for esophageal stenosis following radical surgery for esophageal cancer.

Endoscopy_UCTN_Code_TTT_1AO_2AH

## Notes

Endoscopy E-Videos https://eref.thieme.de/e-videos
*E-Videos* is an open access online section of the journal *Endoscopy* , reporting on interesting cases and new techniques in gastroenterological endoscopy. All papers include a high-quality video and are published with a Creative Commons CC-BY license. Endoscopy E-Videos qualify for HINARI discounts and waivers and eligibility is automatically checked during the submission process. We grant 100% waivers to articles whose corresponding authors are based in Group A countries and 50% waivers to those who are based in Group B countries as classified by Research4Life (see: https://www.research4life.org/access/eligibility/ ). This section has its own submission website at https://mc.manuscriptcentral.com/e-videos .
